# Prevalence and associated factors of anxiety and depression among patients with wounds: An exploratory cross-sectional study in the Taabo health and demographic surveillance system, Côte d’Ivoire

**DOI:** 10.1017/gmh.2026.10133

**Published:** 2026-01-22

**Authors:** Ismaël Dognimin Coulibaly, Bognan Valentin Kone, Bruno Enagnon Lokonon, Didier Yao Koffi, Tanoh Amani Serge Raymond N’krumah, Francis Sena Nuvey, Adja Ferdinand Vanga, Bassirou Bonfoh

**Affiliations:** 1 Université Péléforo Gon Coulibaly, Côte D’Ivoire; 2 Centre Suisse de Recherches Scientifiques en Côte d’Ivoire, Côte D’Ivoire; 3 Institut National de Santé Publique, Côte D’Ivoire; 4 Laboratoire de Biomathématiques et d’Estimations Forestières, Université d’Abomey-Calavi, Benin; 5 Programme National de Lutte contre l’Ulcère de Buruli et des Maladies Cutanées Ulcératives Endémiques, Côte D’Ivoire; 6 Friedrich-Loeffler-Institut Bundesforschungsinstitut fur Tiergesundheit, Germany

**Keywords:** anxiety and depression, wound care, HADS, household-based care, Tabo HDSS, Côte d’Ivoire

## Abstract

**Background:**

Anxiety and depression are common among patients with wounds, impairing healing and quality of life. This study estimated their prevalence and associated factors across community-and referral care facilities in Taabo, Côte d’Ivoire.

**Method:**

An exploratory cross-sectional study included 157 patients aged ≥16 years with wounds, recruited consecutively between October and December 2023. Anxiety and depression were assessed using the Hospital Anxiety and Depression Scale (HADS). Demographic and wound characteristics were collected. Associations were examined using Chi-square or Fisher’s exact tests, and multivariate logistic regression adjusted for age and gender identified independent factors.

**Results:**

Anxiety and depression scores were lowest at household level (6.0 and 5.4) compared to health centre (7.4 and 6.9) and general hospital (9.1 and 9.8). Prevalence was 25.4% and 18.5% at the household level, 49.0% and 55.1% at health centre and 77.4% and 84.9% at the general hospital. Anxiety was independently associated with older age and female gender, while depression was associated to female gender, larger wound size (≥5 cm) and referral-level care.

**Conclusion:**

Early household-based wound care by CHWs was associated with lower prevalence of anxiety and depression. Integrating psychosocial support into wound management, particularly at referral facilities, may reduce the mental health burden.

## Impact statements

Wounds are a growing public health concern in many low- and middle-income countries, where delays in care often lead to chronic conditions that not only impair healing but also affect mental health. This study highlights the often-overlooked psychological burden of wounds by documenting high levels of anxiety and depression among patients receiving wound care in Côte d’Ivoire. Importantly, our findings suggest that patients treated earlier and closer to home by community health workers may experience lower levels of anxiety and depression than those treated at referral facilities. Although exploratory, these results point to the potential dual benefit of community-based wound care: more accessible treatment and a potential reduced psychological burden. For health systems under resource constraints, strengthening community-level care could help limit the need for referral-based treatment, which is both more costly and associated with greater mental health challenges. Our study also underscores the importance of integrating psychosocial support into wound care, particularly in hospitals where chronic wounds are common and the psychological burden is greatest.

## Introduction

In Africa, wounds represent a major public health challenge, stemming from a variety of risk factors linked to the physical environment, daily activities and lifestyle patterns. Limited healthcare infrastructure and economic constraints often result in delayed access to healthcare services, including wound care (Macdonald et al., 2010; Hailemichael et al., 2024). Consequently, many treatable wounds that could heal quickly are either neglected or improperly managed due to long travel distances to health facilities, the cost of treatment, lack of trained personnel and cultural barriers, leading to their progression into chronic conditions (Toppino et al., 2022). These chronic wounds not only lead to severe physical complications but also impose a substantial socio-economic and mental health burden on individuals and communities (Amoakoh and Aikins, 2013; Ogundeji et al., 2023).

The mental health burden in wound care is generally described in the literature using broad terms, such as psychological stress, emotional distress and mood disorders. This includes a wide range of negative effects related to social isolation, stigma, impaired quality of life (Gouin and Kiecolt-Glaser, 2011; Upton et al., 2012; Alderton et al., 2024). In this study, we focus specifically on anxiety and depression, two well-defined mental health disorders. In wound care, anxiety and depression are recognized as some of the most prevalent mental health disorders affecting patients (Sun et al., 2023). Anxiety is characterized by persistent feelings of nervousness, worry and fear, while depression manifests as prolonged sadness, sleep disturbances, loss of confidence and energy and diminished interest in daily life (Renner and Erfurt-Berge, 2017). Both conditions significantly impair the quality of life of patients with wounds. Stressors such as pain and wound odour further exacerbate emotional distress, negatively affecting patients’ well-being and limiting their social interactions (Brenes, 2007; Upton et al., 2012). Prolonged wound duration, particularly in chronic cases, often leads to feelings of exhaustion, resignation and other depressive symptoms, while physical disabilities and altered self-image reduce productivity, increase financial dependence and intensify social isolation (Stienstra et al., 2002; Fino et al., 2019). Beyond clinical and individual-level factors, strong social support has been shown to buffer stress, reduce feelings of isolation and stigma and improve coping mechanisms among individuals with chronic wounds. Conversely, weak family support, social exclusion or community stigma may exacerbate anxiety and depression, reduce treatment adherence and delay healing (Janke et al., 2023; Alia Yadi et al., 2024; Oropallo, 2024).

Emerging evidence further indicates that anxiety and depression may also hinder wound healing by impairing biological processes involved in tissue repair (Cole-King and Harding, 2001; Alderton et al., 2024). For instance, some studies reported that individuals with wounds who exhibit high levels of dysphoria are nearly 3.6 times more likely to experience delayed healing. This occurs because individuals experiencing these emotions may adopt unhealthy behaviours (such as poor diet, reduced exercise or substance abuse) that further impair healing, creating a vicious circle where the wound causes negative emotions that delay healing, which in turn perpetuates further distress (Bosch et al., 2007; Upton, 2014). While much of the available evidence originates from high-income countries, these findings remain relevant in low- and middle income countries contexts, where wound-related stressors – such as stigma, delayed treatment and economic hardship – may further exacerbate these disorders (Koschorke et al., 2022; Adekeye et al., 2023; Okyere et al., 2024).

To mitigate the mental health burden associated with wounds, specifically anxiety and depression disorders, the World Health Organization advocates for early wound management as a critical strategy (Saraceno and Caldas de Almeida, 2022; Yotsu et al., 2023). Early intervention not only enhances healing outcomes but also prevents the progression to chronic wounds and related disabilities (Yotsu et al., 2023). Family and community support systems in this context help to improve adherence to treatment and reduce psychological distress related to wound chronicity (Janke et al., 2023; Shimange et al., 2025).

Across a wide range of health issues, home-based care and early intervention programs have proven highly effective in reducing mental health burdens through preventive and timely care approaches (Saraceno and Caldas de Almeida, 2022; Singh et al., 2022). However, evidence in wound care, particularly in low-income countries, remains scarce. While existing research on chronic diseases and skin neglected tropical diseases (NTDs) in these settings highlights substantial psychosocial harm, including social stigma, emotional distress, discrimination and lower quality of life (Dako-Gyeke et al., 2017; Boukthir et al., 2020; Amoako et al., 2021; Alderton et al., 2024), the prevalence of specific mental health conditions, such as anxiety and depression, among patients with wounds across different levels of healthcare delivery has not been systematically studied.

In this study, we aimed to assess the prevalence and associated factors of anxiety and depression among patients receiving wound care at the household and referral health care levels in Taboo HDSS, Côte d’Ivoire. The results from this study will contribute to providing critical insights into the mental health burden associated with wound care and identify key factors influencing psychological outcomes across different levels of healthcare delivery.

## Material and methods

### Study area

This study was conducted within the Taabo HDSS of Taabo, located in the Tiassalé health district of Côte d’Ivoire, as part of the “Identify & Treat Wounds Early” project implemented since 2019. Data collection took place at Taabo-cité and in the nearby village of Ahondo, situated 15 km away, where previous studies have reported a high prevalence of wounds, including Buruli ulcer (BU) (N’krumah et al., 2017; Toppino et al., 2022). The Taabo HDSS is situated in a rural area of southern central Côte d’Ivoire and encompasses a population of ~61,000 inhabitants across 11,500 households (Koné et al., 2015). Health care infrastructure in the whole Taabo HDSS includes 13 rural primary health care facilities and one general hospital. Economically, the population in this area is predominantly engaged in agriculture and depends largely on seasonal income from coffee and cocoa fields to meet household needs, including health care costs. Access to care, including for wound care, is frequently delayed or limited by financial constraints. Local beliefs about wounds contribute to this delay: so-called in the local language “*kani*” (*e.g.*, traumatic wounds) are often trivialized and self-treated because they are not considered a disease, whereas “*kani teh*” (*e.g.*, BU) are perceived as metaphysical in origin and, therefore, treated by traditional healers. In both cases, such perceptions along with financial barriers hinder timely access to appropriate care within the formal health system, leading to the development of chronic wounds with related disabilities and potential mental health disorders risks (Coulibaly et al., 2023).

### Sociomedical context

This study was conducted within the Taabo HDSS in Côte d’Ivoire, using a community-based wound management model that promotes early detection and treatment of wounds (Toppino et al., 2022). Wound care was organized across the local community (managed by CHWs) and at referral health care levels (health centre and general hospital). Minor, uncomplicated wounds were treated at the local community level by CHWs, while larger, infected wounds or BU (BU) nodules were referred to a health centre for treatment by nurse assistants and nurses. Chronic wounds requiring specialized care, such as antibiotic therapy, debridement, skin grafting, physiotherapy or rehabilitation, were referred to the general hospital for comprehensive, free-of-charge treatment. This multi-tiered approach ensures that patients receive appropriate care tailored to the severity of their condition, optimizing outcomes and resource allocation.

In this study, we defined a wound as a physical injury of the body resulting from infectious, mechanical or metabolic origins. We classified wounds into three main categories. The first category is (i) infectious wounds, which result from pathogens such as bacteria, viruses or fungi that disrupt normal tissue integrity. It includes infectious skin diseases, such as BU, filariasis lymphatic and other common bacterial skin infections, such as erysipelas, rampant in Africa (Stulberg et al., 2002; Velding et al., 2014). The second category is (ii) mechanical wounds resulting from external physical forces (such as abrasions, lacerations, burns or traumatic injuries), and the third one (iii) is metabolic wounds, which often result in internal inflammatory processes (such as autoimmune conditions or chronic inflammatory disorders like vasculitis). It included wounds like diabetic foot ulcers and venous ulcers (Dissemond and Romanelli, 2022; Dumovich and Singh, 2025).

### Study design and participants

We conducted an exploratory, cross-sectional study embedded within a broader wound care implementation project. The study aimed to assess the prevalence of anxiety and depression disorders and identified associated factors with these outcomes among patients receiving wound care at two levels: (i) household-level treatment provided by CHWs and (ii) referral-level care at Ahondo health centre and the general hospital of Taabo. Data were collected using a structured, interviewer-administered questionnaire, which included the Hospital Anxiety and Depression Scale (HADS). Patients were eligible if they were 16 years or older (≥16 years), had a wound (infectious, mechanical and metabolic) and were able to provide written or witnessed informed consent. Exclusion criteria were: (i) inability to provide informed written or witness consent and (ii) patients with dementia or severe psychiatric illnesses. The exclusion of patients with dementia was based on evidence that while the HADS can be feasible in mild dementia, its structural validity is unclear and measurement invariance is poor across levels of cognitive impairment, making interpretation difficult and potentially biased in moderate impairment or severe psychiatric illness (Stott et al., 2017).

Given the exploratory nature of this embedded project component, no formal *a priori* sample size calculation was performed. Instead, the sample size reflects the maximum number of eligible patients identified presenting during the study period (October 2 – December 22, 2023). Participants were recruited consecutively, with all eligible patients invited to take part, helping to minimize selection bias. In total, 157 participants were enrolled: 55 recruited at household care level and 102 from referral facilities (49 from Ahondo health centre and 53 from general hospital of Taabo). The difference in participant numbers between household-level care and referral facilities reflects routine patient presentation patterns and care-seeking pathways during the study period. To allow readers to assess the robustness of the findings despite the absence of a power calculation, results are presented with effect sizes and 95% confidence intervals (CIs).

### Data collection procedures

Data were collected using a structured questionnaire divided into two main sections: (i) clinical and demographic characteristics of patients, and (iii) the HADS to assess symptoms of anxiety and depression disorders. The questionnaire was administered after wound-care sessions. All data were stored using Open Data Kit (ODK) and were anonymized using unique identification codes. The ODK server was password-protected, and access to the data was restricted to authorized members of the research team only. Data were handled confidentially and used exclusively for research purposes.

### Interviewer training and bias reduction

Data collection was conducted by three trained medical personnel: a CHW at the household level, a nurse assistant at Ahondo health centre and a nurse at the general hospital of Taabo. To reduce interviewer bias associated with their dual role in care provision and data collection, all interviewers received specific training on neutral questioning techniques and recording of responses and strictly adhered to the structured questionnaire provided. The data collectors were not provided with detailed information about the specific research objectives.

### Clinical and demographic data

Demographic variables (age and sex) and clinical information, including wound aetiology, size, duration and type of care, were collected across all levels of care. Wound durations were self-reported, while wound size was clinically measured using a ruler. Although self-reported duration may be prone to recall bias, it provided the most feasible estimate in this setting.

#### Assessment of anxiety and depression

The HADS, used to evaluate symptoms of anxiety and depression, is a well-established tool designed to measure these disorders in both healthy and ill populations, as well as in hospital and community settings. It has been validated in diverse languages, countries and contexts, including general practice and community-based studies. The scale consists of 14 items, each rated from 0 to 3, with seven items assessing anxiety and seven items assessing depression, yielding two separate scores (maximum score = 21 for each). The sum of the scores for each subscale is used to determine the severity of anxiety and depression in individuals (Stern, 2014). The tool has demonstrated good psychometric properties across diverse clinical and community populations worldwide, including francophone and African settings (Bjelland et al., 2002; Anye et al., 2023).

### Translation and cultural adaptation of HADS

The questionnaire was administered in French, or when necessary, orally translated into Baoulé and Malinké, which are the two commonly spoken local languages. To ensure consistency, translations were standardized during training sessions, and interviewers were instructed to adhere strictly to agreed-upon wording without any paraphrasing. While no formal back-translation was conducted due to resource constraints, a pilot test with 30 community members and 20 patients across community and referral care levels (health centre and general hospital) confirmed comprehension and cultural appropriateness. The pilot sample size was determined pragmatically, based on feasibility and the need to include participants from both community and referral care settings and from the main language groups. A total of 50 participants (30 community members and 20 patients) was considered sufficient to identify major comprehension or translation issues, in line with common practice for pilot testing of survey instruments in exploratory studies (Teresi et al., 2022; Bujang et al., 2024). Although the tool has not been formally validated in this specific rural HDSS setting, the measures implemented helped to reduce potential misinterpretation. From the exploratory perspective of this study, the approach was considered sufficient to generate preliminary insights. Future research should prioritize full cultural and linguistic validation to enhance the scale’s applicability in this context.

### Data analysis

Data were analysed using R software. Descriptive analyses summarized demographic, clinical and HADS variables. Chi-square and Fisher’s exact tests assessed associations between anxiety, depression and patient characteristics. Since HADS scores were not normally distributed, Kruskal–Wallis tests compared scores across care levels. Where appropriate, variables were categorized or transformed. Multivariate binary logistic regression identified predictors of anxiety and depression as binary response variables. The independent variables considered in the models were the type of wound, wound size, level of care and wound duration. The models were adjusted for age and gender as confounders. The Hosmer–Lemeshow goodness-of-fit test showed *p* > 0.05, indicating that the considered models fit the data well. Adjusted odds ratios (AORs) and CIs were reported for predictors included in the models. Analysis of deviance of the fitted models was performed to assess the significance of individual predictors. *P* < 0.05 was considered statistically significant. Moreover, there was no missing data.

The HADS scores were interpreted as follows: a subscale score of >8 out of a possible 21 indicates significant symptoms of anxiety or depression, while a score of ≤8 suggests the absence of such symptoms (Rishi et al., 2017). This threshold provided a clear framework for categorizing patients’ mental health status and analysing the prevalence and associated factors of anxiety and depression in the study population. This threshold is widely used in international validation studies (Bjelland et al., 2002; Olssøn et al. 2005) and has recently been applied in francophone African settings (Anye et al., 2023).

## Results

### Sociodemographic and wound characteristics of patients

Table 1 presents the wound characteristics, including the distinct patterns in wound aetiology and chronicity across different levels of care, and sociodemographic profiles of the patients. The majority of participants were adult females (*n* = 82, 52.2%). At the community level, all patients (*n* = 55) presented with acute mechanical wounds (<1 month duration) mainly related to trauma. By contrast, referral health care facilities managed more heterogeneous and chronic cases. At the Ahondo health centre, wounds were mostly mechanical (*n* = 39, 79.6%) with a small proportion of infectious (*n* = 5, 10.2%) and metabolic cases (*n* = 5, 10.2%), such as BU, erysipelas and diabetic foot ulcers. At the general hospital of Taabo, chronic wounds predominated, including infectious wounds such as BU, erysipelas (*n* = 18, 34.0%), mechanical wounds related to trauma and snakebite (*n* = 24, 45.3%) and metabolic wounds related to diabetes (*n* = 11, 20.8%). Larger wounds (*n* = 47, ≥5 cm) and longer durations (*n* = 61, ≥1 month) were observed at referral health care facilities.

**Table 1.** Sociodemographic and wound characteristics of patients

Variables	Local community care (*n* = 55)	Referral health care facilities (*n* = 102)	
		Health centre (*n* = 49)	General hospital (*n* = 53)
**Age (years), mean**	*32.1*	*37.6*	*41.7*
16–21	10 (18.2)	10 (20.4)	4 (7.6)
22–35	28 (50.9)	14 (28.6)	15 (28.3)
36–54	16 (29.1)	16 (32.7)	22 (41.5)
≥55	1 (1.8)	9 (18.4)	12 (22.6)
**Sex**			
Male	25 (45.5)	22 (44.9)	28 (52.8)
Female	30 (54.5)	27 (55.1)	25 (47.2)
**Type of wounds**			
Infectious wounds	0 (0.0)	5 (10.2)	18 (34.0)
Mechanical wounds	55 (100.0)	39 (79.6)	24 (45.3)
Metabolic wounds	0 (0.0)	5 (10.2)	11 (20.8)
**Size of wounds**			
<5 cm	55 (100)	43 (87.8)	12 (22.6)
≥5 cm	0 (0.0)	6 (12.2)	41 (77.4)
**Duration of wounds**			
≤1 month	52 (94.5)	39 (79.6)	4 (7.6)
≥1 month	3 (5.5)	10 (20.4)	49 (92.4)

### Prevalence of anxiety and depression disorders among patients

Table 2 presents the proportions of potential anxiety and depression cases, along with the corresponding mean scores, among patients treated at different levels of care. Among those receiving care at the household level with CHWs, 25.5% (*n* = 14) exhibited symptoms of anxiety and 18.2% (*n* = 10) showed symptoms of depression. In contrast, at the referral health care facilities, nearly half of the patients reported symptoms of anxiety (*n* = 24, 49.0%) and over half reported depression (*n* = 27, 55.1%) at the health centre. These proportions were even higher at the general hospital of Taabo, where nearly three-quarters (*n* = 41, 77.4%) presented with anxiety and more than three-quarters (*n* = 45, 84.9%) with depression symptoms. Mean scores for both anxiety and depression increased significantly across levels of care (*p* < 0.001, Kruskal–Wallis test).

**Table 2.** HADS scores and prevalence of anxiety and depression symptoms across community and referral care health care facilities

	Community care (*N* = 55)	Referral care facilities (*n* = 102)		*P*-value
		Health centre (*N* = 49)	General hospital (*N* = 53)	
Anxiety				
Mean score	6.0	7.4	9.1	*p* < 0.001***
Possible case of anxiety (HADS cut-off score ≥ 8)	14 (25.5%)	24 (49.0%)	41 (77.4%)	
Depression				
Mean score	5.4	6.9	9.8	*p* < 0.001***
Possible case of depression (HADS cut-off score ≥ 8)	10 (18.2%)	27 (55.1%)	45 (84.9%)	

Disaggregated descriptive analyses showed the distribution of prevalence of anxiety and depression across demographic factors and wound characteristics.

Patients with a wound duration of ≥1 month (*n* = 62, anxiety = 67.7%, depression = 67.7%) were more likely to exhibit symptoms of anxiety and depression compared to those with a wound duration of <1 month (*n* = 95, anxiety = 36.8%, depression = 33.7%). Similarly, patients with large wound sizes ≥5 cm (*n* = 47, anxiety = 74.5%, depression = 85.1%) were more likely to display symptoms of depression compared to those with wound sizes <5 cm (*n* = 110, anxiety = 40.0%, depression = 43.6%). Patients with infectious wounds (*n* = 23, anxiety = 78.3%, depression =82.6%) and metabolic wounds (*n* = 16, anxiety = 68.8%, depression = 75.0%) were more likely to have more symptoms of anxiety and depression compared to patients with mechanical wounds (*n* = 118, anxiety = 42.4%, depression = 48.3%). Patients receiving care through household-based community services (*n* = 55, anxiety = 25.5%, depression = 18.2%) exhibited lower symptoms of anxiety and depression compared to those treated at referral care facilities (*n* = 102, anxiety = 68.6%, depression = 62.7%). As for sociodemographic characteristics, gender differences were also observed, with females experiencing a higher level of anxiety and depression (*n* = 75, anxiety = 60.0%, depression = 65.3%) than males (*n* = 82, anxiety = 41.5%, depression = 47.6%). Younger patients aged 16–21 years (*n* = 24, anxiety = 45.8%, depression = 54.2%) and 22–35 years (*n* = 57, anxiety = 35.1%, depression = 63.2%) experienced lower levels of anxiety compared to older patients aged 36–54 years (*n* = 54, anxiety = 59.3%, depression = 55.6%) and those aged 55 years and above (*n* = 22, anxiety = 72.7%, depression = 81.8%).

### Association between anxiety, depression and demographic and wound characteristics of patients

Table 3 shows a bivariate association between anxiety, depression and patients’ demographic and clinical characteristics using Chi-square or Fisher’s exact tests as appropriate. Due to the cross-sectional design, these findings indicate associations, not causality. Regarding sociodemographic characteristics, age was significantly associated with anxiety (*p* = 0.04) and depression (*p* < 0.001). Similarly, sex was also associated with both anxiety (*p* = 0.02) and depression (*p* = 0.003). Wound characteristics showed clear associations with mental health outcomes. Wound size (<5 cm = 110, ≥5 = 47), duration (<1 month = 95, ≥ 1 month = 62), type of wounds (infectious wounds = 23, mechanical wounds = 118, metabolic wounds = 16) and level of care (household level care = 55, health centre = 49, general hospital = 53) were associated with anxiety and depression.

**Table 3.** Bivariate associations between anxiety, depression and patients’ characteristics

	Modalities	Anxiety		*P*-value	Depression		*P*-value
		No anxiety	Anxiety		No depression	Depression	
**Sociodemographic characteristics**							
**Age**		78	79	0.04	69	88	<0.001
**Sex**	Male	48	34	0.02	43	39	0.03
	Female	30	45		26	49	
**Wound characteristics**							
**Type of wounds**	Infectious wounds	5	18	0.002	4	19	0.003
	Mechanical wounds	68	50		61	57	
	Metabolic wounds	5	11		4	12	
**Wound size**	<5 cm	66	44	<0.001	62	48	<0.001
	≥ 5 cm	12	35		7	40	
**Level of care**	Local community care	41	14	<0.001	45	10	<0.001
	Referral care	32	70		38	64	
**Wound duration**	<1 month	60	35	<0.001	63	32	<0.001
	≥ 1 month	20	42		20	42	

Significance level: *** *p* < 0.001, ** *p* < 0.01, * *p* < 0.05.

### Factors independently associated with anxiety and depression among patients with wounds

The multivariable logistic regression indicated that age and gender were independent risk factors for anxiety (Table 4). Each additional year of age increased the odds of anxiety by 1.03% (AOR = 1.03, 95% CI: 1.03–1.06, *p* = 0.04), while females had higher odds compared with males (AOR = 0.38, 95% CI: 0.18–0.80, *p* = 0.01). Borderline associations were observed for level of care and wound duration. Patients treated at referral facilities were more likely to report anxiety than those treated in the community (AOR = 2.35, 95% CI: 0.97–5.85, *p* = 0.06), and wounds lasting ≥1 month were associated with higher odds compared with wounds <1 month (AOR = 2.84, 95% CI: 0.92–8.99, *p* = 0.07). No significant associations were found for wound size or wound type.

**Table 4.** Multivariable binary logistic regression of factors associated with anxiety

Characteristic	Adj. OR	95% CI	*P*-value
**Age**	1.03	1.00–1.06	0.04
**Gender**			
Female	1		–
Male	0.38	0.18–0.80	0.01
**Type of wound**			
Metabolic wounds	1		–
Infectious wounds	1.40	0.25–7.81	0.70
Mechanical wounds	1.00	0.20–4.48	1.00
**Wound size**			
<5 cm	1		–
≥5 cm	1.37	0.41–4.55	0.60
**Level of care**			
Local community	1		–
Referral care	2.35	0.97–5.85	0.06
**Wound duration**			
<1 month	1		–
≥1 month	2.84	0.92–8.99	0.07

Significance level: *** *p* < 0.001, ** *p* < 0.01, * *p* < 0.05.

Concerning the depression, the multivariable logistic regression analysis showed that gender, wound size and referral care were independently associated with anxiety and depression (Table 5). Females had significantly higher odds of depression compared with males (AOR = 0.44, 95% CI: 0.20–0.93, *p* = 0.03). Larger wounds (≥5 cm) were linked to higher odds of depression than smaller wounds (AOR = 6.37, 95% CI: 1.50–30.67, *p* = 0.02). Patients treated at referral facilities had increased odds of depression compared with those managed at the household level (AOR = 3.03, 95% CI: 1.27–7.48, *p* = 0.01).

**Table 5.** Multivariable binary logistic regression of factors associated with depression

Characteristic	Adj. OR	95% CI	*P*-value
**Age**	1.02	0.99–1.05	0.26
**Gender**			
Female	1		–
Male	0.44	0.20–0.93	0.03
**Type of wound**			
**Metabolic wounds**	1		–
Infectious wounds	4.28	0.64–36.10	0.14
Mechanical wounds	2.78	0.49–17.51	0.25
**Wound size**			
<5 cm	1		–
≥5 cm	5.93	1.52–30.67	0.02
**Level of care**			
Local community	1		–
Referral care	3.03	1.27–7.48	0.01
**Wound duration**			
<1 month	1		–
≥1 month	1.37	0.40–4.52	0.60

Significance level: *** *p* < 0.001, ** *p* < 0.01, * *p* < 0.05.

## Discussion

This study is part of the “*Identify and Treat Wounds Early*” project, implemented since 2019 in Taabo HDSS in Côte d’Ivoire. The project employed a transdisciplinary and participatory approach, engaging communities and equipping CHWs with standardized kits and training to recognize and treat small wounds at the household level, while referring more complex cases to higher levels of care (health centres and general hospitals).

Our findings reveal that around 25% of patients treated at the household level by CHWs exhibited symptoms of anxiety and 18% for depression. These proportions were lower compared to patients treated at referral care facilities, where over 50–80% showed symptoms of anxiety and depression. This disparity can be attributed to differences in wound characteristics between the two care levels. Most community-level wounds were small and acute (<1 month, <5 cm), while referral facilities managed large and chronic wounds (≥1 month, ≥5 cm). Chronicity and wound size are known to exacerbate pain, distress and impaired quality of life, thereby increasing risk of anxiety and depression (Klis et al. 2014; Upton, 2014; Olsson et al. 2019). In low-income countries, Daré et al. (2019) reported a pooled prevalence of 36.6% for anxiety and/or depression among individuals with chronic physical diseases, with a threefold higher risk compared to those without chronic conditions.

In these settings, several contextual factors may also contribute to wound chronicity and related psychological burden. Delayed access to biomedical care, often due to reliance on traditional practices, can lead to wound-worsening and systemic infections (Dawer et al. 2025). Socio-economic disadvantages, such as job insecurity, precarious employment and lower levels of education, are key factors contributing to both wound chronic and mental health disorders. These conditions may restrict individuals and households’ access to healthcare services for wound care and hinder their capacity to cope with life stressors, thereby increasing their vulnerability to mental illness (Garchitorena et al., 2017; Alzahrani et al., 2024). Social stigma resulting from malodorous sequelae or disabilities associated with wounds often leads to exclusion, discrimination and reduced self-esteem, exacerbating, therefore, psychological distress (Tuwor et al., 2024).

All these factors not only compromise mental health but also impair wound healing (Cole-King and Harding, 2001; Guo and DiPietro, 2010; Gouin & Kiecolt-Glaser, 2011). This underscores the need to embed mental health care as a core element of wound management, with routine screening for anxiety and depression during wound assessments, basic psychosocial support delivered by trained wound-care staff and integrated care plans that track psychological status alongside healing progress.

Beyond estimating prevalence, our study identified independent factors associated with anxiety and depression. In multivariate logistic regression analysis, anxiety was independently associated with older age and female sex and showed borderline associations with longer wound duration and being treated at referral facilities. Depression was significantly associated with wound size and referral care.

Older patients were more likely to report anxiety, aligning with studies showing age-related anxiety due to social isolation and poor health (Sikström et al., 2023). Female patients exhibited higher odds of anxiety and depression compared to males. Similar findings have been reported in other chronic disease populations, where female sex was associated with greater anxiety and depression (Khullar et al., 2016; Al-Ayed et al., 2021). These results highlight the need for gender-sensitive and age-sensitive psychosocial support and interventions.

Regarding wound-related factors, large wounds were independently associated with depression, and longer duration showed borderline links with anxiety, supporting previous findings that wound severity is a key driver of psychological distress (Dantas et al. 2022). The observed associations emphasize that comprehensive wound management should integrate psychosocial care, with early intervention aimed at preventing wound progression and mitigating mental health consequences.

Finally, referral-level care was independently associated with depression, showing high odds among patients treated at these levels compared to those managed at the household level. While this association cannot establish causality, this pattern suggests that timely community-based wound care interventions may help to alleviate the psychological burden of chronic wounds. These findings are consistent with those of Shipowick et al. (2025) and Toppino et al. (2022), who reported reduced suffering when CHWs provided accessible, home-based wound care. Embedding CHWs within the community at the household level to provide timely wound care may offer dual benefits – improving clinical outcomes while supporting psychological well-being.

To our knowledge, this study is the first to assess anxiety and depression in wound patients in Côte d’Ivoire and provides a foundation for further research into the psychosocial burden of wounds and skin-related NTDs. A key strength of this study is its comparative design, which assessed anxiety and depression across community care levels and referral health care facilities. This multi-level perspective moves beyond hospital-based studies by showing that early intervention at the community level may be associated with lower prevalence of psychological distress, while also highlighting the important unmet need for psychosocial support in referral facilities. Nevertheless, the study has some limitations. As a cross-sectional study, the observed associations do not confirm definitive evidence or establish causal relationships of household-based wound care improving mental health outcomes. Consequently, future research should pursue large-scale longitudinal and intervention studies to strengthen the evidence base, assess causality and enhance the generalizability of the results. Lastly, the absence of an Ivorian-specific version of the HADS may reduce cultural sensitivity in capturing anxiety and depression symptoms. Developing a locally adapted version of the HADS could improve its applicability in future studies.

Despite these limitations, our study offers preliminary evidence to guide health authorities in exploring integrated care models that address both physical and psychological needs. The associations observed suggest the value of embedding routine mental health screening and support into wound care services, with CHWs playing a pivotal role in early identification and referral. At the national level, integrating these components into wound management protocols could foster more holistic patient care.

## Conclusion

Our findings suggest that early identification and treatment of wounds at the household level may be associated with lower prevalence of anxiety and depression among patients receiving wound care. The substantial burden of anxiety and depression observed in referral facilities, where chronic wounds are managed, underscores the need to promote not only early detection of wounds but also the integration of psychological support into wound care protocols. Future research should pursue large-scale longitudinal and intervention studies to strengthen causal evidence and improve generalizability.

## Data Availability

The data that support the findings of this study are available from the corresponding author (IDC) upon reasonable request.
