# Supplementary material for: Prevalence and associated factors of anxiety and depression among patients with wounds: An exploratory cross-sectional study in the Taabo health and demographic surveillance system, Côte d’Ivoire
Source: Glob Ment Health (Camb). 2026 Jan 22;13:e29. doi: 10.1017/gmh.2026.10133 (PMC12914471; doi:10.1017/gmh.2026.10133)
Supplement: Peer review history

# Editor report: Recommendation: Prevalence and associated factors of anxiety and depression among patients with wounds: An exploratory cross-sectional study in the Taabo health and demographic surveillance system, Côte d’Ivoire — R1/PR2

**DOI:** 10.1017/gmh.2026.10133.pr2

**Affiliations:** Psychology, UNISA, South Africa

Dear Dr Coulibaly,

Thank you for your revised manuscript. We have reached a decision regarding your submission. I am pleased to inform you that your manuscript has now been accepted for publication.

Thank you for submitting your interesting and important work to the Cambridge Prisms: Global Mental Health.

Kind regards,

Dr Limkile Mpofu 

Handling Editor (Cambridge Prisms: Global Mental Health)

# Editor report: Decision: Prevalence and associated factors of anxiety and depression among patients with wounds: An exploratory cross-sectional study in the Taabo health and demographic surveillance system, Côte d’Ivoire — R1/PR3

**DOI:** 10.1017/gmh.2026.10133.pr3

**Affiliations:** London School of Hygiene & Tropical Medicine, United Kingdom of Great Britain and Northern Ireland

No accompanying comment.
